# Adapting a brief mindful breathing intervention for self-management of distress in advanced cancer patients: the RESOLVE-i study

**DOI:** 10.1186/s12904-026-02148-3

**Published:** 2026-05-28

**Authors:** Carole A. Paley, Max Henderson, Sheila Freeman, Cornelia Reyes Acosta, Lucy Ziegler, Emma J. Chapman

**Affiliations:** 1 University of Leeds, Academic Unit of Palliative Care, Leeds Institute of Health Sciences, Worsley Building Level 10, Clarendon Road, Leeds, LS2 9NL UK; 2 University of Leeds, Leeds Institute of Health Sciences, Worsley Building Level 10, Clarendon Road, Leeds, LS2 9NL UK; 3 Patient and Public Engagement Representative, Leeds, LS2 9NL UK

**Keywords:** Distress, Palliative cancer care, Mindful breathing

## Abstract

**Background:**

Psychological distress is common among patients with advanced cancer and can be a barrier to effective symptom management. Despite national guidance, psychological support in UK palliative care remains limited. Existing interventions are frequently delivered by untrained staff lacking confidence. Adapting evidence-based interventions offers an efficient strategy to improve care. This study describes the adaptation of a brief mindful breathing intervention, originally developed in Malaysia, for self-management of distress by patients with advanced cancer in the UK.

**Methods:**

Using Moore et al.’s ADAPT framework, we followed four stages:

Step 1: A systematic review (PROSPERO: CRD42022311729) established a rationale for adapting a brief mindful breathing intervention for distress in advanced cancer.

Step 2: Semi-structured interviews with healthcare professionals (HCPs) explored acceptability, perceived utility, and integration into routine care.

Step 3: A series of interviews with patients and carers informed the cultural and contextual iterative adaptation of the mindful breathing intervention and the development of accessible self-management resources.

Step 4: A feasibility study was designed to assess acceptability, implementation, and to generate pilot data.

**Results:**

The systematic review supported the effectiveness of mindful breathing, though evidence found was context-specific to Malaysian clinical settings. HCPs endorsed integration into routine care but noted time constraints and concerns about information accessibility. Feedback from patients and carers informed several adaptations for self-management, including simplified language, inclusive imagery, removal of prescriptive instructions (e.g., breathing through the nose), and development of low-literacy resources. A video animation, infographic, and HCP training package were created to support implementation.

**Conclusions:**

This stakeholder-informed adaptation resulted in a self-management mindful breathing intervention tailored for patients with advanced cancer in the UK. The intervention is now ready for feasibility testing and represents a scalable, resource-efficient strategy to enhance psychological support in palliative care, in line with NHS goals for community-based self-management.

**Supplementary Information:**

The online version contains supplementary material available at 10.1186/s12904-026-02148-3.

## Background

Psychological distress can be a major barrier to effective management of physical symptoms in patients with cancer [1, 2]. Poorly controlled pain, breathlessness and fatigue are common in patients with cancer. These symptoms, coupled with loneliness and psychological distress amongst both patients and carers have been associated with greater unscheduled out-of-hours healthcare use [3]. Over half of palliative care patients experience distress, often when their emotional needs are not well managed [4]. This rarely reaches “mental illness” thresholds, so psychiatric interventions are not indicated. However, existing palliative care approaches are not always effective at alleviating distress [5, 6]. Current psychological support in palliative care is often inadequate [6], despite national guidance recommending better support [7]. Our national survey showed interventions are often provided by untrained staff who lack confidence in delivery [8]. A better intervention for distress could facilitate the management of physical symptoms and reduce episodes of unplanned care use in the wider health service.

The adaptation of existing evidence-based healthcare interventions rather than the development of entirely novel approaches can offer a more efficient use of time and resources [9, 10]. When performed systematically, adaptation can be effective, provided that contextual differences between the original and target settings are carefully considered and that the efficacy of the intervention is preserved across these contexts [10]. The ADAPT guidelines, developed by Moore et al., offers a structured, stepwise approach to guide this process, emphasising the importance of identifying both contextual similarities and distinctions [10, 11]. Effective adaptation further requires an iterative, methodologically rigorous approach that actively engages relevant stakeholders to ensure both contextual relevance and fidelity to the intervention’s core components [12].

Our recent systematic review identified several studies utilising brief mindful breathing interventions to support distressed cancer patients [13]. These interventions—typically five minutes in duration—were conducted in Malaysia, delivered face-to-face to cancer patients by healthcare professionals, and embedded in distinct religious, cultural, and belief-based contexts [14, 15]. Other studies have used mindful breathing for management of physical symptoms [16–19]. A 5-minute mindful breathing script originally developed in a Malaysian pilot study by Beng TS, et al., [14] and used in a subsequent study by the Guan et al. [16]. This script was selected for adaptation to a United Kingdom (UK) setting for cancer patients with distress.

We describe the process of adapting this mindful breathing intervention, employing the ADAPT guidelines [11], for implementation as a supported self-management strategy to for cancer patients with distress in the UK (the RESOLVE-i study). The stages used in the adaptation process are described and provide detail on an iterative process employed through engagement with stakeholders including patients, carers and healthcare professionals within primary care and hospice settings (consultants, general practitioners, hospice clinical nurse specialists, staff nurses and allied health professionals). Most people in the UK receiving specialist palliative care live at home and receive hospice outpatient care and homecare. With the shift from ‘hospital to community’ a key theme of the recent 10-year health plan for England and the development of the neighbourhood health service this is an ideal framework in which to introduce mindful breathing as a supported self-management intervention [20].

### Aims and objectives

The RESOLVE-i study aimed to investigate whether a brief mindful breathing intervention for distress, originally developed in Malaysia, could be adapted for self-management by patients with advanced cancer in the UK.

The objectives of the study were to:

1. Adapt a 5-minute mindful breathing intervention for self-directed use in a UK population with advanced cancer.
2. Determine how to effectively integrate mindful breathing into usual patient care.
3. Design a further study to test the feasibility of implementing introduction of the intervention as part of usual care.

### Research approvals

The RESOLVE-i study was approved by the Health Research Authority and the London - Camden and Kings Cross Research Ethics Committee (Ref: 24/LO/0484). All methods were carried out in accordance with the principles of the Declaration of Helsinki. Informed consent (written or recorded) was obtained from all participants immediately prior to the interview.

## Methods

**Fig. 1:**
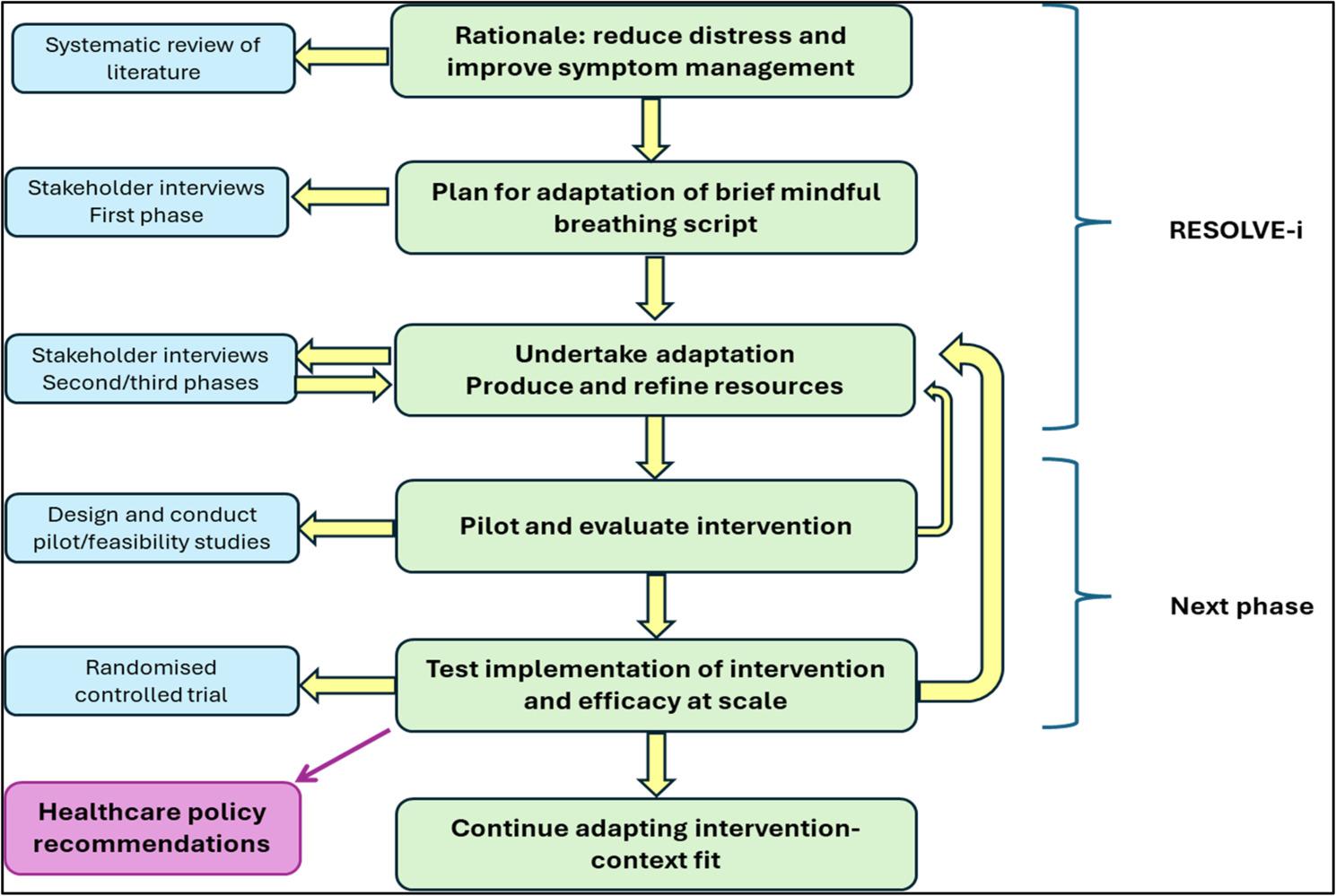
Application of ADAPT guidelines in the RESOLVE-i study

Using the steps described in the ADAPT guidance [11] the following methodology was used (also see Fig. 1).

### Methodology step1: developing a rationale for the intervention

This key stage in the adaptation process requires the development of an evidence-based rationale for adapting the intervention for use in a new context.

#### Systematic review of literature

Previous research has identified that psychological distress is prevalent in patients living with cancer and it can hinder effective management of physical symptoms [1]. Several systematic reviews had identified that psychological interventions, including mindfulness, were effective in alleviating distress [21–24]. However, the evidence was less certain on which interventions specifically provided relief of cancer-related distress.

We therefore conducted a systematic review of literature to determine the efficacy of non-pharmacological interventions for distress in in patients with active cancer excluding cancer survivors [13]. This was registered on the PROSPERO database (number CRD42022311729).

Current UK Government policy and National Institute for Health and Care excellence (NICE) guidelines [7, 20, 25] were also explored to determine how a self-help intervention would fit within a UK health system. The systematic review of literature was used to underpin the planning stage of the adaptation.

### Methodology step 2: planning for the adaptation

#### Adapting to a new context

Adapting an intervention for use in a different setting requires an understanding of the new context and an understanding of stakeholder perspectives. Differences in religion, culture and healthcare provision between Malaysia and the UK mean that the intervention might not be readily transferrable. Malaysia has a two-tiered healthcare system: a heavily subsidised public system and a well-developed private system. Malaysia is known for its ethnic diversity. Islam is the predominant religion, although Buddhism, Hinduism, and Christianity all play significant roles. Shamanism and traditional healing practices are also part of this diverse society.

Therefore, as part of the preparation to adapt the brief mindful breathing intervention for a UK cancer population, an understanding of the perceived need for, and acceptability of, the intervention amongst healthcare professionals (HCPs), the practicalities of introducing it and the perspectives of patients and carers was needed. From the outset, patient and public representatives were embedded within the research team.

We conducted interviews with stakeholders as follows:

1. Semi structured recorded interviews with HCPs within primary care (GP practice teams) and hospice care across the Leeds and Bradford region.
2. Semi structured recorded interviews and focus groups with patients and carers from a hospice inpatient and outpatient population in three iterative phases.

#### Participants and recruitment process

Eleven HCPs involved with the care of patients with advanced cancer were identified and purposively recruited across two hospice sites in the North of England and from GP practice teams within West Yorkshire. They were selected according to the following criteria:

- Qualified HCPs including medical doctors/consultants, nurses, allied health professionals or complementary therapists.
- Directly involved in the palliative care of cancer patients within hospice or GP practice settings.

Thirteen inpatients and outpatients and their carers were purposively recruited to the study, based on the following criteria:

- Patients having palliative care for cancer, and their carers/relatives.
- Age ≥ 18 years.
- Attending hospices as inpatients or outpatients.
- Selected by hospice staff to take part in the study on a voluntary basis.

All participants gave informed written (or audio recorded) consent and demographic information was recorded.

#### Semi structured interviews

Immediately following informed consent, semi-structured interviews were conducted either in-person or via video link and were audio recorded onto an encrypted device. All recordings were transcribed and pseudonymised by assigning each participant with an identification code. Any other identifiable information was removed from the transcripts.

Interviews with HCPs explored the acceptability of the intervention idea, how this might fit within each clinical role, the resources/time required for delivery and practical considerations for introducing it. Interviews with patients and carers considered attitudes to mindful breathing, its acceptability and aspects around how it might be introduced and delivered, including any resources and formats that patients would find useful.

**Fig. 2:**
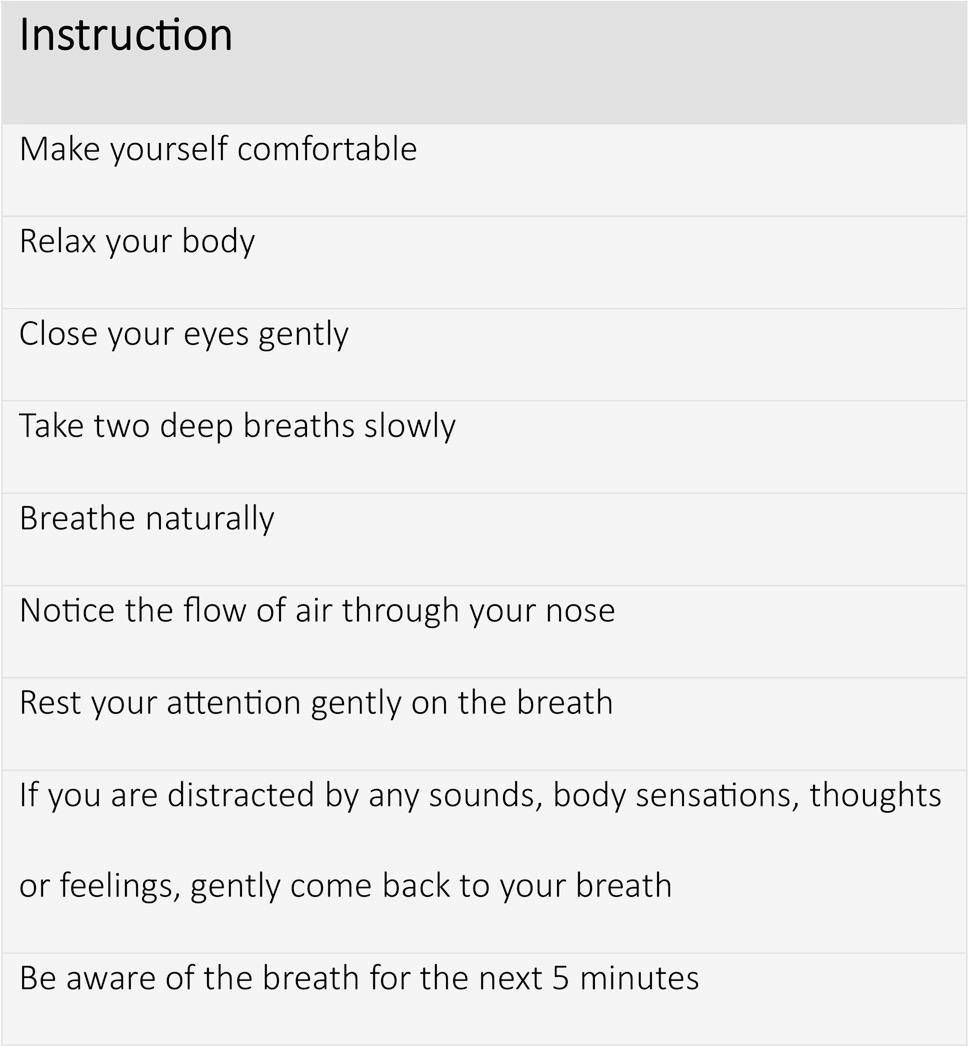
Original 5‑minute mindful breathing script, adapted from Beng et al. and Guan et al., [originally published under a CC BY‑NC‑SA 4.0 licence] [14, 16]

The interviews with patients and carers were conducted in three iterative phases, starting with the original brief mindful breathing script (Fig. 2) [14, 16].

In phase one, participants were introduced to the original script and at each subsequent phase, adaptations were made to the script, and these were presented at the next phase of interviews. Finally, resources to promote engagement and delivery were produced and presented to patients and carers.

### Data analysis

Framework analysis, as described by Sekhon, et al. [26], was used to code and analyse data. Two researchers (CP and EC) coded the data at each phase independently. Discrepancies were discussed (with the aid of a 3rd researcher (LZ) if necessary) and a consensus coding agreed. Responses were grouped under seven broad headings: affective attitude, burden, ethicality, intervention coherence, opportunity costs, perceived effectiveness and self-efficacy. Key themes extracted from the data were used to inform changes to the prototype mindful breathing script using an iterative process throughout each phase of interviews.

### Methodology step 3: undertaking the adaptation

#### Identifying facilitators and barriers to implementation

In the first round of interviews with stakeholders we identified enablers and potential barriers to implementation of the mindful breathing intervention and discussed how any constraints could be overcome. For example, HCPs were concerned with aspects of delivery such as acceptability to patients, time constraints within patient-practitioner consultations and accessibility of information.

#### Co-design of intervention and resources

The final mindful breathing script was developed in three stages using an iterative process. The final script was used as a template to develop an animated 90-second video and printed infographic, to support delivery of the intervention. These resources were co-designed following the analysis of our phase 2 interviews, alongside our expert patient panel and a commercial company. The co-design of resources involved several stages of development and reviews of an animated video and infographic before the final versions were produced. These were presented to patients and carers in the third phase of interviews.

#### Testing the adaptation of the mindful breathing intervention

Preliminary testing of the prototype intervention was carried out in the phase three interviews. Feedback from patients and carers was collected in individual interviews and focus groups. Interviews were audio-recorded and conducted either in-person or remotely and transcribed.

#### Implementation of the mindful breathing intervention

Following adaptation, a training package was designed to help HCPs introduce the intervention to patients and carers, including instructions on PowerPoint slides which could be delivered as a short presentation.

### Methodology step 4: piloting and evaluation of adapted intervention

The final refined version of the script and the resources developed during the study were used to inform the design of a feasibility study which is described in below in the results Section 4.

## Results

The results of each stage of the ADAPT process are summarised below and in Fig. 4.

Self-reported demographic details of participants are shown in Tables 1 and 2.

Participants were predominantly female (HCPs and patients/carers) and all were white British. The HCPs were from a range of professions, both within hospice settings and in primary care. In the patient/carer group, six participants were carers. Participants in this group had a range of occupations and educational status varied.

**Table 1:** Demographic information HCPs

Characteristic	Participants (*n* = 11)
Gender	Female: 9
	Male: 2
Healthcare provider	GP surgery: 4
	Hospice: 7
Professional role	GP: 3
	Con: 1
	CNS: 1
	RNS: 1
	AHP: 2
	SN: 1
	CT: 1
	PN: 1
Years’ experience in current role	Average: 9.6 yrs Range: 3–20 years

*GP *general practitioner, *Con *Hospice consultant, *CNS *Clinical nurse specialist, *RNS *Respiratory nurse specialist, *AHP *Allied Health Professional, *SN *Staff nurse (hospice), *CT *Complementary therapist, *PN *Practice nurse

**Table 2:** Demographic information patients and carers

Characteristic	Category	Patient (*n* = 7)	Carer (*n* = 6)
Gender	Male	4	2
	Female	3	4
Age	30–39		2
	40–49		
	50–59	1	1
	60–69	2	2
	70–79	3	
	80–89		1
	90+	1	
Ethnicity	White	7	6
	Other	0	0
Cancer type	Lung	1	N/A
	Bowel	1	
	Chest (unknown)	1	
	Breast	1	
	Brain	1	
	Unknown	2	
Current or previous occupation	Retired banker	1	
	Retired truck H&S instructor	1	
	Retired market researcher	1	
	Retired farmer	1	
	Operations Manager	1	
	Sales representative	1	
	Lecturer/Teacher		3
	Medical supplies work		1
	Electrical retailer		1
	Retired (unknown)		1
	Unknown	1	
Educational status	Professional qualification	1	N/A
	O-levels	2	
	Further education	1	
	City & Guilds	1	
	Unknown	2	

### Results step 1: developing a rationale for adapting the intervention

Our systematic review provided rationale for using a brief mindful breathing intervention to help patients with cancer manage distress [13]. Other studies also show that brief sessions (5–30 min) of mindful breathing improved distress [14, 27]. However, these were all carried out in Malaysia and therefore might not be translatable to a UK context. Religious affiliation has been shown to affect acceptability of mindfulness-based interventions [28]. Malaysia is predominantly an Islamic country with Buddhism being the second most identified religion. Healthcare in Malaysia is public and privately funded and routinely provides a mixture of Western and traditional Eastern medicine. In our time- and resource-pressured health services, evidence-based and well-designed approaches that can be used as self-management interventions, with minimal resources and training have an important role.

### Results step 2: planning for the adaptation

This stage was designed to help understand stakeholder perspectives and involved HCPs, service leads, patients and carers.

#### Healthcare professionals

Eleven HCPs were interviewed using a semi-structured interview schedule (supplementary information). Overall, HCPs supported the adaptation of the intervention, and some were already using variations of mindful breathing with their patients.

Key learning from the interviews have been divided into responses from primary care practitioners and hospice staff. Tables 3 and 4 respectively show the comments made by the interviewees and the actions taken to adapt the intervention accordingly.

#### Primary care clinicians

1. GPs felt that time constraints during consultations might prevent them from being able to deliver the intervention, so they preferred something which was easy and quick to explain and teach.
2. Some GPs also worried that patients might be negative because they were expecting medication rather than a complementary, non-pharmaceutical approach.
3. Finding information on the intervention or remembering what it entailed during a time-limited consultation was a worry. Provision of simple resources for patients would help.
4. It would be useful to provide patients with some instruction before sending them home to try the intervention.

#### Hospice staff

1. Some of the Clinical Nurse Specialists thought that introducing the intervention was a better fit within the roles of staff nurses, allied health professionals (AHPs) or complementary therapists.
2. Time pressures were less of a burden for HCPs working in hospice settings.
3. There was more opportunity to find somewhere suitable to deliver the intervention within hospice settings.
4. Hospice staff welcomed the opportunity to deliver the intervention to both patients and their carers.
5. A one-page handout or poster or a short video would help with delivery and engagement.

#### Patients and carers

Thirteen patients and carers were recruited to the study. Semi-structured interviews and focus groups were carried out (see supplementary information).

From the patient and carer perspective, responses were largely positive. Overall, both patients and carers were open to engaging with the intervention and understood that it would support them in managing their distress, thus enabling them to access other interventions more readily.

Key learning points from the interviews with patients and carers were:

1. Some patients had previously tried mindfulness or engaged in similar techniques without formally recognizing them as such.
2. Patients and carers felt that it would be useful to have some basic instruction to introduce the intervention.
3. Introduction to the intervention in a group setting might be useful.
4. Something to prompt and remind them to use the intervention would be beneficial.

### Results step 3: undertaking the adaptation

#### Identifying facilitators and barriers to implementation

##### Interviews with healthcare professionals

The coded transcripts of the interviews were used to assess the acceptability of the mindful breathing intervention, ethical issues, burden, opportunity cost, intervention coherence and self-efficacy, within the Framework of Acceptability [26].

Using the original mindful breathing script, healthcare professionals considered the suitability of introducing this to patients within the constraints of a consultation. HCPs identified elements of the script which might not be appropriate for their patient populations and suggested adaptations which are summarised in Table 3:

**Table 3:** Adaptation responses to HCPs

Healthcare professional comment (summarised)	Adaptation response
Closing the eyes is not appropriate if patients need to look at instructions.	Instruction to close the eyes was removed from the mindful breathing script.
Instruction to nose-breathe is not appropriate for some patients.	Any reference to breathing through the nose was removed from the script.
Breathless patients would struggle to regulate duration of breaths by counting.	The script did not include any instructions to count the duration of breaths.
Easily accessible information is needed which can be given or sent to patients.	The mindful breathing resources and instructions are readily accessible and easily understood in a variety of formats.

##### Interviews with patients/carers: phase 1

In the phase 1 interviews patients and carers were introduced to the original Malaysian 5-minute mindful breathing intervention script [16]. This was positively received by most patients. Two patients were initially negative when mindful breathing was suggested, but through the course of the interview they realised that mindful breathing was something they had already tried and found useful. This suggests that the term ‘mindfulness’ might not be appropriate for some patients. Responses to patient and carer interviews are listed in Table 4:

**Table 4:** Adaptation responses to patients and carers: phase 1

Patient/carer comment (summarised)	Adaptation response
Relaxing can be difficult, and there could be constant interruptions or noise.	The potential for interruptions or background noise was acknowledged, with an instruction to bring the focus back to the breath.
Instruction to close the eyes was not well received.	References to closing the eyes were removed.
Inhaling through the nose or counting breaths felt unnatural.	All content involving counting breaths or inhaling through the nose were removed.
Written instructions would be helpful.	A written script was developed.

##### Interviews with patients and carers: phase 2

During the phase two interviews, changes to the script were made to reflect the comments in phase one, and the modified script was presented to patient and carers. The modified script was acceptable to patients and carers. A key suggestion from this second phase was that alternative resources would be useful and improve accessibility.

The comments from patients and carers in the phase 2 interviews and our adaptation responses are listed in Table 5.

**Table 5:** Adaptation responses to patients and carers: phase 2

Patient/carer comment	Adaptation response
It would be useful to have a video to help patients follow the mindful breathing script.	A 90-second video animation was produced to improve accessibility.
Written instructions are important.	A written script was produced and to improve accessibility, an infographic was designed, based on the video animation and the written script.
There is a need for resources to be accessible.	The video and infographic were developed to represent neutral gender and ethnicity, and the provision of written, pictorial and video resources increases accessibility.

##### Preparation of resources

**Fig. 3:**
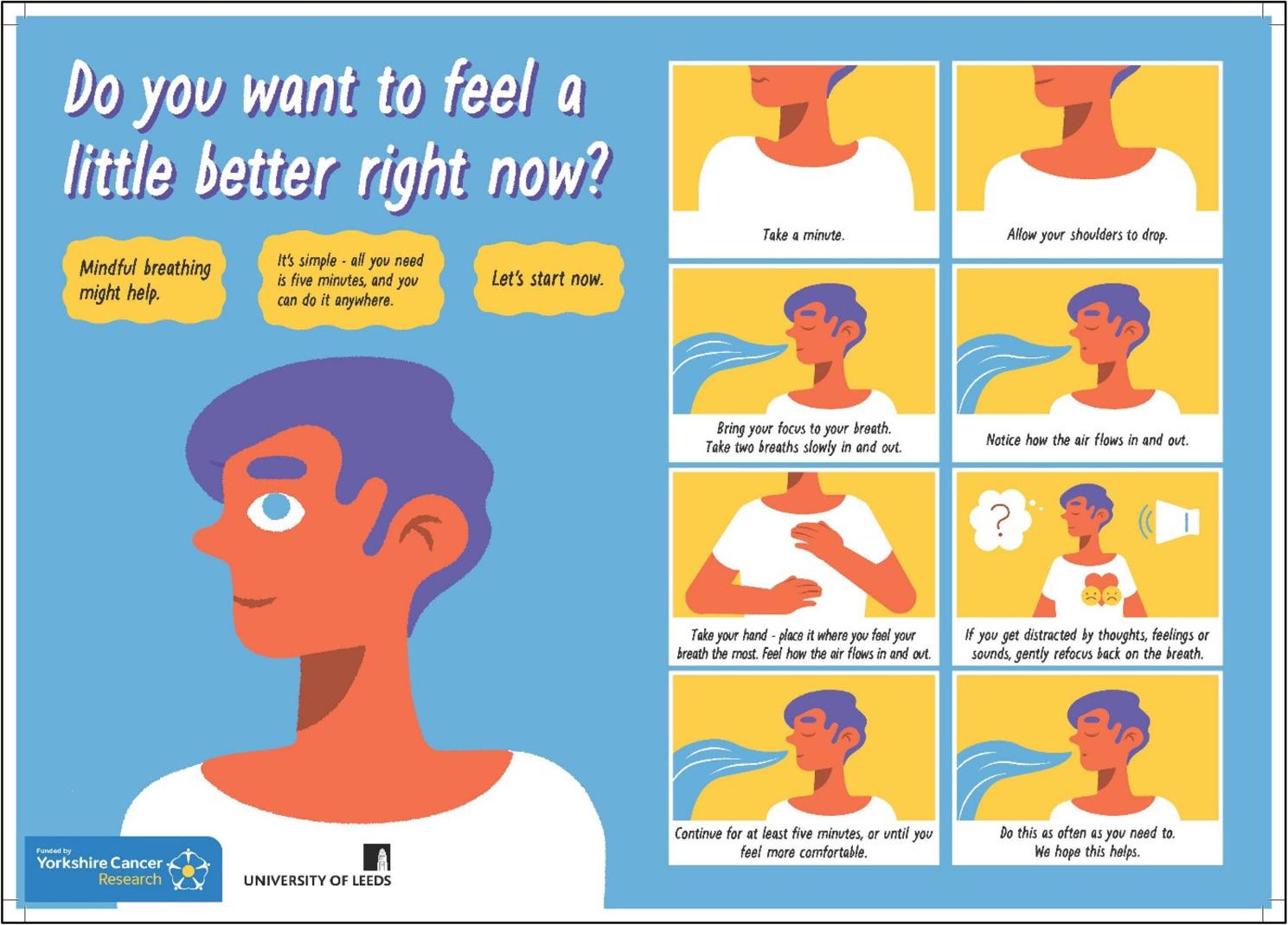
Infographic for mindful breathing (Designed by Nifty Fox Creative, 2025)

Prior to phase three, resources were professionally co-designed with patient and clinician input for maximum accessibility and inclusivity. These resources included a 90-second video animation showing the mindful breathing instruction sequence, and an accompanying infographic (Fig. 3).

Key aspects of these resources are as follows:

1. The printed infographic allows access by those without technological ability/internet access.
2. The ability to understand written or spoken English is not necessary to follow and learn the mindful breathing procedure on both infographic and animation.
3. Hosting the animation on YouTube will allow access on a variety of devices without the need for a specific application.
4. Animation subtitles improve accessibility for those with deafness.
5. Simple, relaxing audio voiceover script improves accessibility for those with visual impairment.
6. Reading age assessment (Gunning Fog index = 4.6) of audio and script indicates this would be understood by a person with primary school level education and above.
7. The character in the infographic and animation is of neutral gender and ethnicity.

The resources were co-designed and reviewed by our patient panel to further refine the content. Additional adaptations included:

1. Changes to the text, e.g., “Do you want to feel a little better…”, “it’s simple”, “take a minute”. These instructions were in patient-friendly and easily understood language designed to simply introduce the intervention.
2. No specific anatomical features were included (e.g., drawing of the lungs) to avoid unpleasant association with X-rays.
3. Changes were made to the figure in the video and infographic to remove reference to nose-breathing.
4. The figure was presented as gender-fluid and of no specific ethnicity.
5. The voiceover for the video was chosen specifically for its relaxing timbre, and the timing of the instructions was refined to provide a pace which felt unhurried and relaxed for the panel members.

##### Interviews with patients and carers: phase 3

The final phase of patient/carer engagement involved introducing the video and infographic as a resource to seven patients and carers who had not been exposed to the study resources before. They were asked to review the resources and discuss feasibility, implementation and engagement.

Participants were shown the animation which was presented as a 90-second video, together with the accompanying infographic, and asked to comment. These final comments and our responses are shown in Table 6.

**Table 6:** Adaptation responses to patients and carers: phase 3

Patient/carer comment	Adaptation response
The colours are too bright – can they be toned down.	A decision was made to keep the colours as they were because only one carer commented on this.
The animated figure has its eyes closed when breathing. Could this be changed or just have it as an option.	The feasibility study, which will follow on from the RESOLVE-I study, will gather data on the acceptability of the resources. Currently, eye-closure is optional. The animated figure could be changed in future.
The instruction to place the hands on the chest might be problematic for some participants.	This is currently an optional instruction. It will be assessed for acceptability in the following feasibility study.
The video is very short. Could there be an option to having it on a loop? Possibly with an instruction to “stay with me” before continuing.	This suggestion will be taken forward to our feasibility study with a view to extending the duration if desired.

**Fig. 4:**
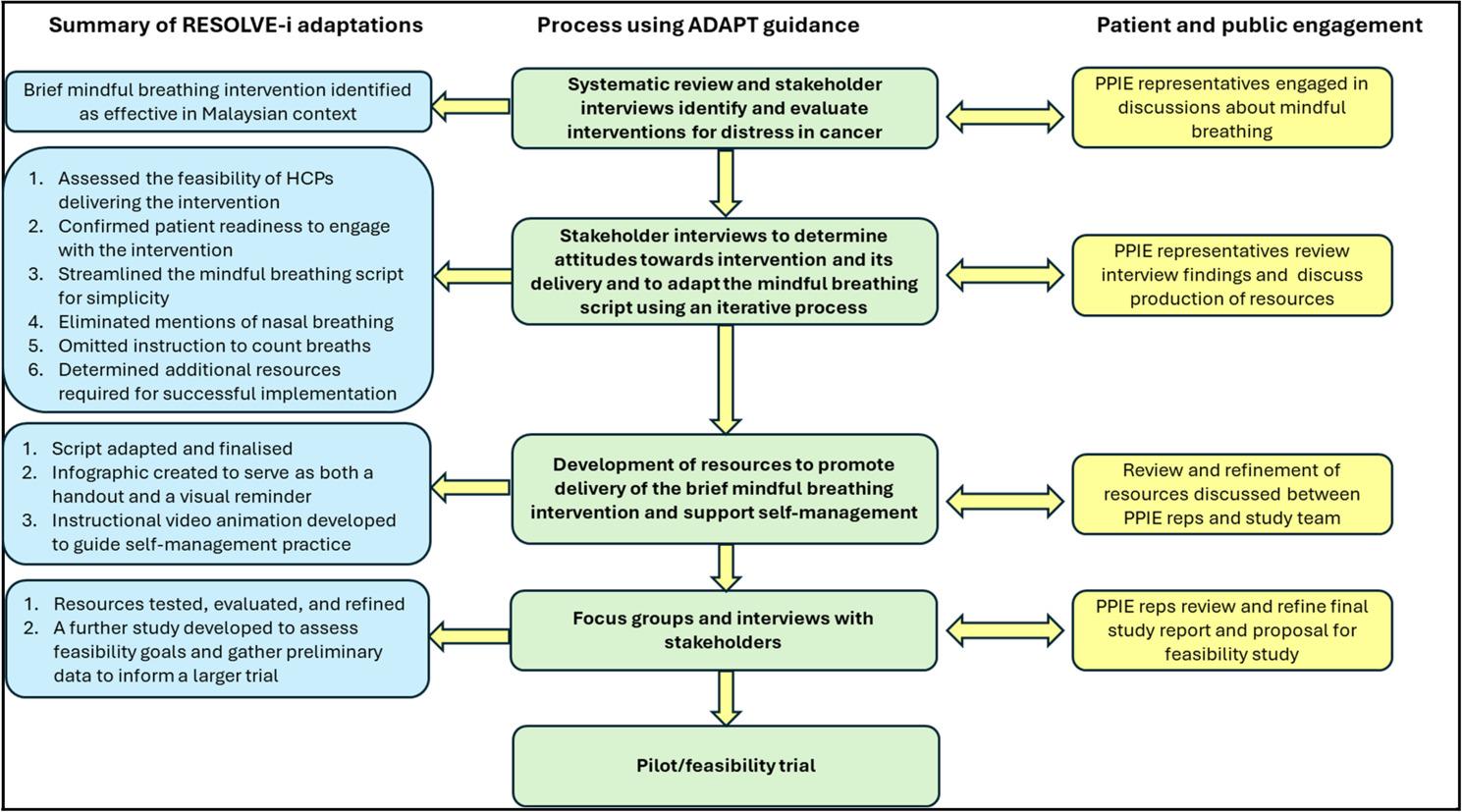
Adaptation process flowchart

A diagrammatic summary of the adaptation process is shown in Fig. 4.

#### Training package for healthcare professionals

We were able to identify enablers and barriers to introducing and implementing the mindful breathing intervention within the structure of care settings or GP practices. Although there was an enthusiasm for introducing the brief mindful breathing intervention, we highlighted a need for staff education and training and for the provision of resources and information to facilitate delivery to patients.

Following the final refinements to the resources accompanying our adapted mindful breathing intervention, a training package was developed for healthcare professionals as a tool to help them introduce the intervention to patients. This consisted of printed information and set of PowerPoint slides to be used alongside the video animation and infographic. The training package explained the rationale and evidence supporting the intervention and explained how it could be introduced within clinical practice.

### Results step 4: piloting and evaluation of adapted intervention

A single-arm, non-randomised feasibility study (INTEGRATE study), was designed to pilot the adapted intervention with the following specific objectives:

1. Determine if it is feasible and acceptable to introduce a self-directed mindful breathing intervention for distress to advanced cancer patients.
2. Assess the validity of progression to a randomised pilot trial.
3. Optimise the protocol for a randomised pilot and definitive trial of effectiveness.

Key uncertainties to be addressed are recruitment to target, retention, reasons for attrition, sample diversity, fidelity, burden, opportunity cost, adherence, suitability and burden of outcome measures, alongside patient and carer perceptions. The mindful breathing technique will be introduced to a sample of 32 patients at four hospice sites by HCPs at hospice outpatients’ groups or in individual consultations. Patients will be given written instructions, infographic and animation access and encouraged to practice at home, when needed for distress, for 6 weeks. Brief, validated, assessment measures (Five facet mindfulness questionnaire-short form, Distress Thermometer, and Integrated Palliative care Outcome Scale) will be completed at baseline, 3, 6 weeks after intervention introduction. In addition, semi structured, recorded interviews will explore acceptability, reasons for adherence and attrition from the perspectives of patients, carers and HCPs. For the purposes of recruitment, it will be necessary for participants to have a life expectancy of at least 8 weeks to avoid attrition due to death.

## Discussion

Involving patient, carer and HCP stakeholders in the adaption of the intervention and embedding patient and public representatives within the research team highlighted important contextual aspects that could not have been identified by academic researchers alone. From the HCP perspective we gained a better understanding of the practicalities and competing priorities of introducing an additional intervention during consultations or groups with a range of professionals. From the patient and carer perspective we were able to explore attitudes and opinions about this approach to mindful breathing. Ultimately, co-adapting the intervention with representatives of key stakeholders has enabled developed of an intervention ready to progress to feasibility and acceptability testing.

All the HCPs interviewed found the intervention acceptable and felt it would be a useful addition to usual care. Some of the hospice clinical nurse specialists felt that it might not be their role to introduce the intervention, although ideally this might change in time. It was suggested that within the hospice, complementary therapists, physiotherapist and staff nurses may be more suitable to introduce mindful breathing. Both patients and HCPs thought introducing the intervention within a group setting would be useful for promoting patient engagement.

Time pressures were particularly relevant to primary care staff. The GPs were particularly keen for resources which could be delivered within the time constraints of a 10-minute consultation and would also act as a prompt for patients. Some hospice staff were able to spend longer with each patient but depending on their role may have had competing priorities.

Patients and caregivers expressed favourable opinions regarding the intervention, with some reporting prior usage of similar techniques, albeit without formally identifying them as such. Some patients initially viewed the intervention as something associated with ‘alternative’ lifestyles. This underpins our intention that both staff training and patient information materials must highlight the scientific basis for the use of this intervention and that proof of efficacy exists.

During the process of adapting the mindful breathing script for use in the UK, the language and instructions used were simplified and made less prescriptive. A key part of these adaptations was ensuring that patients could self-manage this intervention for use when needed, using the resources developed.

The short and simple animation of the script and the design of the infographic were chosen and adapted according to patient preference. These offered written, pictorial, animated and audio instructions to maximise inclusivity.

### Study limitations

In palliative care research, recruitment and attrition of patient participants can be high due to fluctuations in health, feelings of being overwhelmed and gatekeeping by family or health professionals [29]. These barriers can, to some extent, be overcome by allowing flexibility in recruitment strategies. However, recruitment to our study was still time-consuming and subject to last-minute changes.

Although our study aim was to adapt an intervention for individuals with distress, experiencing distress was not an inclusion criterion for participants. Therefore, the opinions of the patients and carers sampled may not represent our final target population.

All participants were aware of the topic of the interview before agreeing to take part which may naturally have selected for those with an interest in non-pharmacological approaches to symptom management. We noted that several HCPs and one bereaved carer expressed personal interest and involvement in mindful breathing techniques.

Finally, we were aware that the ethnic diversity of participants did not reflect the population of the local areas used for the research. We recruited primary care HCP participants from deprived areas of Leeds and Bradford. Recruiting hospices serve differing socioeconomic populations and one is based in an area of socioeconomic deprivation. Nevertheless, the interviewees all identified as white British. This aligns with other research which has identified that barriers still exist for the provision of palliative and end-of-life care for ethnic minority groups [30].

## Conclusions and implications for further research

This paper provides a contextual example of how the ADAPT guidance, as described by Moore, et al. [11] has been used to adapt a brief mindful breathing intervention for distress, for self-management use in a UK setting by patients with advanced cancer. Adapting the intervention for self-management provides greater potential for widespread use in a range of settings with reduced resource burden. Ultimately, it is our intention that this will allow more patients and carers to benefit from using the intervention to manage distress. A feasibility trial which will determine practical aspects of conducting a large, phase III study to test the efficacy of the intervention, is planned.

## Supplementary Information

Supplementary Material 1.

Supplementary Material 2.

## Data Availability

The datasets used and/or analysed during the current study are available from the corresponding author on reasonable request.

## Abbreviations

AHP: Allied Health Professional
CNS: Clinical Nurse Specialist
CT: Complementary Therapist
GP: General Practitioner
HCP: Healthcare Professional
NHS: National Health Service
PN: Practice Nurse
RNS: Respiratory Nurse Specialist
SN: Staff Nurse
UK: United Kingdom
