# Supplementary material for: Adapting a brief mindful breathing intervention for self-management of distress in advanced cancer patients: the RESOLVE-i study
Source: BMC Palliat Care. 2026 May 28;25:218. doi: 10.1186/s12904-026-02148-3 (PMC13411096; doi:10.1186/s12904-026-02148-3)
Supplement: Supplementary file 1 — Supplementary Material 1. [file 12904_2026_2148_MOESM1_ESM.docx]

| **Topic guideline for healthcare professionals** | | |
| --- | --- | --- |
| Collection of informed consent | | |
| Welcome and introduction to the aims of RESOLVE i.  Recap of our previous work on the role of distress in symptom management. | | |
| **Introduction** | Can you tell me a little about your role?  How many years have you worked here?  How many years have you worked in palliative care? | |
| **Introduce intervention prototype** | | |
|  |  | Example questions |
| **Affective attitude** | How an individual feels about the intervention | First thoughts on this mindful breathing, intervention. |
| **Burden** | Perceived amount of effort to introduce the intervention | How would you introduce the intervention during a patient consultation? Other ways it could be introduced? |
| **Ethicality** | Is the intervention a good fit with your value system | To what extent does introducing the intervention align with the remit of your role and the objectives of a patient consultation? |
| **Intervention Coherence** | How well do you understand the intervention and how it works | How confident would you feel explaining why we think this may help with symptom management to a patient |
| **Opportunity costs** | The extent to which benefits, values, profits (and time) might be given up to introduce the intervention | Do you forsee any disadvantages of introducing the intervention? |
| **Perceived Effectiveness** | The extent to which the intervention is perceived likely to achieve its purpose | Would you expect this intervention to be effective t? Are there any intended or adverse effects that concern you and if so, what are they?? |
| **Self-efficacy** | Confidence that the individual can perform the necessary behaviours to introduce the intervention | How confident would you feel to introduce the intervention and answer any queries about it? |
| Any other thoughts or comments? | | |
| Thank you | | |
