# Supplementary material for: Adapting a brief mindful breathing intervention for self-management of distress in advanced cancer patients: the RESOLVE-i study
Source: BMC Palliat Care. 2026 May 28;25:218. doi: 10.1186/s12904-026-02148-3 (PMC13411096; doi:10.1186/s12904-026-02148-3)
Supplement: Supplementary file 2 — Supplementary Material 2. [file 12904_2026_2148_MOESM2_ESM.docx]

| **PHASE 1** | **Understanding patient and carer perspective on a mindful breathing intervention.** | |
| --- | --- | --- |
| **Theme** | **General questions** | **Prompt items** |
| **Introduction** | Introduce Research – Exploring the addition of a mindful breathing intervention for psychological distress to optimise symptom management for patients living with advanced cancer | - Introduce self - Explain confidentiality, length of interview/group, nature of discussion - What we are going to cover - Any question - Start recording - Obtain consent - Complete demographic form - Participants invited to introduce themselves |
| **Mindfulness approaches for psychological distress** | Are you aware of, or have you been introduced to mindfulness approaches | - Prompt: examples - If you have tried mindfulness, was it helpful? - If it was helpful, can you explain how it helped? - What did you particularly like/dislike about it? - If you were aware of it but had not used, why? |
| **Introduction to the original professional facilitated iteration of a mindful breathing** | We are interested in a short mindful breathing intervention, and we would like to know what you think about it. | - Describe the mindful breathing script as used in Malaysian studies and explain what data has shown regarding effects on distress and other symptoms.   The script will be demonstrated, by a researcher and participants invited to follow it if they wish.   - First thoughts? (brainstorming activity) - Is there anything you would change about this approach?   Prompt: how clear were instructions.?  Any problems encountered?  Benefits? |
| **Closing** | What we will be doing next with what we have learnt today. | Reminder about right to withdraw consent in next 2 weeks and how to contact researchers.  Thank you for your participation |

| **PHASE 2** | **How should a mindful breathing intervention be adapted for self-management use?** | |
| --- | --- | --- |
| **Theme** | **General questions** | **Prompt items** |
| **Introduction** | Introduce Research – Exploring the addition of a mindful breathing intervention for psychological distress to optimise symptom management for patients living with advanced cancer | - Introduce self - Explain confidentiality, length of interview/group, nature of discussion - What we are going to cover - Any question - Start recording - Obtain consent - Complete demographic form   Participants invited to introduce themselves |
| **Introduction to the original professional facilitated iteration of a mindful breathing** | We are interested in developing a short mindful breathing intervention that people could use at home to help when they are feeling distressed. | - Describe the mindful breathing script as used in Malaysian studies and explain what data has shown regarding effects on distress and other symptoms.   The script will be demonstrated, by a researcher and participants invited to follow it if they wish. |
| **How to develop the intervention for use in a self-management approach** | How might we develop this intervention for people to use at home? | - Do you think people would be able to use this approach without a healthcare professional beside them? - What problems might you expect? - Which healthcare professionals (in hospice or elsewhere) might you expect to tell you about this kind of approach? - When and where would you like to learn about it? - What sort of format might be useful for the instructions if you were to do this at home? - Is the wording OK? - Discuss pros and cons of different formats e.g. written instructions, prompt card, audio, online, carer training, App etc? |
| **Closing** | What we will be doing next with what we have learnt today. | Reminder about right to withdraw consent in next 2 weeks and how to contact researchers.  Thank you for your participation |

| **PHASE 3** | **Development of a prototype mindful breathing intervention for self-management use in the UK.** | |
| --- | --- | --- |
| **Theme** | **General questions** | **Prompt items** |
| **Introduction** | Introduce Research – Exploring the addition of a mindful breathing intervention for psychological distress to optimise symptom management for patients living with advanced cancer | - Introduce self - Explain confidentiality, length of interview/group, nature of discussion - What we are going to cover - Any question - Start recording - Obtain consent - Complete demographic form   Participants invited to introduce themselves |
| **Background** | Describe the mindful breathing script as used in Malaysian studies and explain what data has shown regarding effects on distress and other symptoms. | Discuss what we have done so far and what other participants told us. |
| **Mindful breathing self-management intervention prototype(s)** | Introduce and discuss the self-management intervention prototype(s) | - Participants to be introduced to intervention by researcher and then try independently if wish. - Discussion of e.g. ease in following instructions, clarity, practicality. - Explore according to the framework of acceptability. - Affective attitude – how they feel about the intervention. - Burden- perceived effort to use the intervention. - Ethicality - Is the intervention a good fit with your values/beliefs? - Coherence – How well do you understand the intervention and how it works? - Opportunity costs – how much time would be taken? - Perceived effectiveness – do you think it would help someone who is distressed? - Self-efficacy – do you think that patients would be able to use the intervention? - What would make it more likely that someone would use the intervention? Further suggestions for amendments? |
| **Closing** | What we will be doing next with what we have learnt today. | Reminder about right to withdraw consent in next 2 weeks and how to contact researchers.  Thank you for your participation |
